# Establishing Doping Limits for ZnGa_2_O_4_ for Ultrawide-Band-Gap Semiconductor Applications

**DOI:** 10.1021/acsami.5c19146

**Published:** 2025-12-09

**Authors:** Romain Claes, Alexander G. Squires, David O. Scanlon

**Affiliations:** School of Chemistry, 83415 University of Birmingham, Edgbaston, Birmingham B15 2TT, U.K.

**Keywords:** ZnGa_2_O_4_, ZGO, wide band gap, TCOs, oxide, defects, transport, renormalization

## Abstract

ZnGa_2_O_4_ is an ultrawide-band-gap oxide with promising applications as a transparent conductor and a deep-UV electronic material. Despite this, their transport and doping limits remain poorly defined. Here, we present a comprehensive computational study combining hybrid density functional theory, density functional perturbation theory, and advanced transport modeling. We show that ZnGa_2_O_4_ exhibits a dispersive conduction band minimum with a low effective mass (0.27 *m*_0_), supporting phonon-limited electron mobilities approaching 500 cm^2^ V^–1^ s^–1^. However, impurity scattering dominates across experimentally relevant carrier concentrations, limiting the achievable mobility to values consistent with state-of-the-art measurements. Temperature-dependent band gap renormalization due to electron–phonon coupling is quantified and found to be strongly asymmetric between the conduction and valence bands, an effect that is essential to reproduce experimentally observed intrinsic carrier concentrations (∼9 × 10^19^ cm^–3^). Defect calculations reveal that Ga/Zn antisites pin the Fermi level, driving degenerate *n*-type conductivity under typical growth conditions, while a *p*-type behavior is unlikely due to deep acceptor levels and polaron formation. Screening of extrinsic dopants demonstrates limited potential for further carrier enhancement, with most substitutions yielding high formation energies or deep traps. These findings establish the intrinsic and extrinsic doping limits of ZnGa_2_O_4_, highlighting both its potential as a deep-UV transparent conductor and the challenges for further performance optimization.

## Graphical abstract

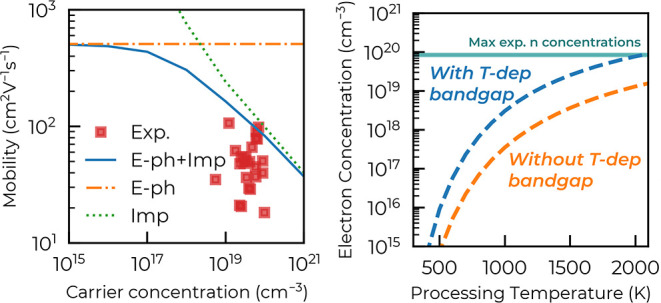

## I Introduction

Ultrawide-band-gap (UWBG) oxides are attracting increasing attention for applications spanning transparent conductors, power electronics, and deep-UV optoelectronics. Their chemical stability, wide band gaps, and favorable electronic properties make them highly versatile platforms for next-generation devices.^1^ β-Ga_2_O_3_ has been at the forefront of this field, demonstrating excellent breakdown strength and mobility,^2–5^ but related oxides are now emerging as complementary candidates.

Among these, ZnGa_2_O_4_ (zinc gallate, abbreviated as ZGO) crystallizes in the normal spinel structure (*Fd*3̅*m*), with Zn^2+^ on tetrahedral sites and Ga^3+^ on octahedral sites, as shown in Figure 1. The resulting framework of edge-sharing GaO_6_ octahedra yields a dispersive conduction band similar to β-Ga_2_O_3_, suggesting the potential for high carrier mobility.^6^ Together with its wide direct band gap (∼5 eV), ZnGa_2_O_4_ is a promising candidate for *n*-type transparent conducting oxide (TCO) deep-UV applications.^7,8^ Reports of *p*-type conductivity exist, but the limited hole density and evidence of small-polaron formation make ambipolar operation unlikely.^9–13^

**Figure 1:**
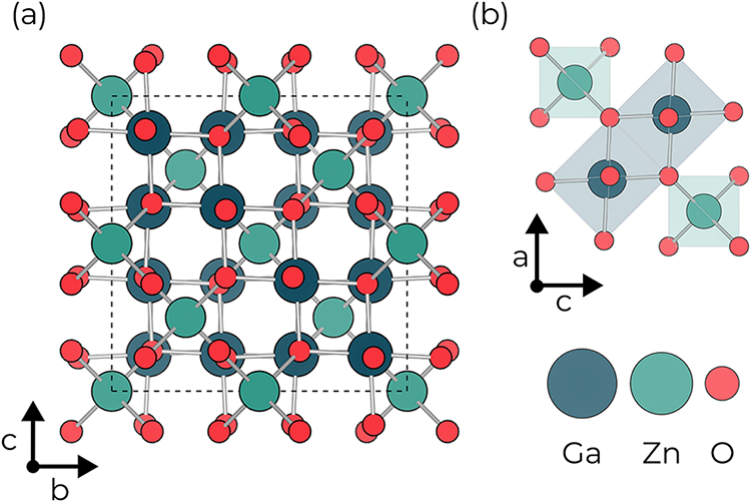
(a) Crystal structure of ZnGa_2_O_4_ (*Fd*3̅*m*) with (b) the corresponding crystal connectivity of the ZnO_4_ tetrahedra and GaO_6_ octahedra.

Beyond electronic transport, ZnGa_2_O_4_ has demonstrated bright blue luminescence, tunable emission with transition-metal or rare-earth dopants,^14–18^ and a long afterglow, as well as utility in gas sensing and photocatalysis.^19–21^ This multifunctionality underscores its technological relevance but also highlights the need to clarify its fundamental transport and doping limits.

Here, we provide a comprehensive computational study of the electronic transport, temperature-dependent band gap renormalization, and defect chemistry of ZnGa_2_O_4_. Using a combination of hybrid density functional theory and first-principles transport calculations, we establish the intrinsic mobility limits, quantify the role of phonon and impurity scattering, and show how temperature-dependent band gap narrowing reconciles intrinsic defect chemistry with experimentally observed carrier concentrations. We further evaluated the feasibility of extrinsic doping strategies and demonstrated the limitations imposed by antisite defect formation. Together, these insights define the potential and limitations of ZnGa_2_O_4_ as a deep-UV transparent conductor and UWBG electronic material.

## II Computational Methodology

The different density functional theory (DFT) (and density functional perturbation theory, DFPT) calculations were performed using either the Vienna ab initio simulation package (Vasp ) code^22,23^ (hybrid functional band structure, Amset^24^ inputs and defects) or Abinit^25,26^ (phonon dispersions, iterative transport calculations, and temperature-dependent band gaps). The plane-wave cutoff and **k**-point grid were converged for both softwares, resulting in a cutoff of 450 eV for Vasp and 45 Ha for Abinit and a **k**-point grid of 5 × 5 × 5. Properties based on DFPT, i.e., phonon dispersions, phonon-limited mobility, temperature-dependent band gaps, and ionic dielectric constant calculations, were performed using the Perdew–Burke–Ernzerhof for solids (PBEsol)^27^ exchange–correlation (XC) functional within the generalized gradient approximation (GGA). The remaining calculations, i.e., band structures, Amset inputs, and defect calculations, were done using the hybrid PBE0 functional.^28^ GGA and hybrid relaxations of the structure lead to lattice parameters of 8.34 and 8.33 Å, respectively. Both are in close agreement to experimental measurements (8.33 to 8.37 Å).^4,29,30^

Charge transport calculations were split into two contributions: electron–phonon scattering and impurity scattering. The former was computed using the iterative solution of the Boltzmann transport equation (IBTE), as described in refs 31,32 and implemented in the Abinit code. The calculations were performed with the inclusion of the dynamical quadrupoles,^33^ even if it does not seem to be of crucial importance in this specific case (see Figure S2). We assume a convergence of the IBTE mobility when three successive grids are within a 5% range, which was obtained with 100 × 100 × 100 **k**/**q**-meshes in the case of ZnGa_2_O_4_. The convergence study can be seen in the Supporting Information. On the other hand, a Brooks–Herring-based model was used for the impurity-limited mobility, as implemented in Amset software. The different inputs used in this model are described in the Supporting Information. Finally, the phonon- and impurity-limited results were combined following Matthiessen’s rule ( $\frac{1}{\mu_{e\text{−}\mathrm{ph}}}+\frac{1}{\mu_{e\text{−}imp}}$ with μ_e–ph_ and μ_e–Imp_ being the contributions of electron–phonon and electron–impurity scattering, respectively), in a similar way as in previous works.^34,35^

Abinit was also used to compute the temperature-dependent band gap of ZnGa_2_O_4_ within the nonadiabatic Allen–Heine–Cardona (AHC) formalism and the harmonic approximation.^36,37^ In this work, we include only the contribution due to the e–ph interaction, leaving the temperature/volume (e.g., thermal expansion) and anharmonic contributions for future works.^36,38^ We discussed the implication of the absence of these contributions in Section 4. The e–ph self-energy can be written as the sum of the frequency-dependent Fan–Migdal term and the static Debye–Waller term,^37,39,40^ both obtained from DFPT. Quasiparticle (QP) corrections from e–ph coupling are evaluated in the on-the-mass-shell (OTMS) approximation, rather than by solving the linearized QP equation. We choose OTMS approximation because a recent work indicates that it yields results closer to those obtained with more advanced cumulant expansion treatments of the e–ph spectral function.^41^ Finally, the Sternheimer approach was used to accelerate the convergence with respect to the number of bands.^40^ Within this setup, convergence was achieved with 80 bands (10 empty bands) and a **q**-mesh of 40 × 40 × 40 (Figure S3).

Point defects were modeled using the supercell approach. To minimize the interactions between periodic defects, a supercell of 84 atoms was used to maintain a >10 Å distance between periodic images, as generated using the doped package.^42^ The ShakeNBreak approach^43,44^ was used to identify the ground-state defect structures of each intrinsic and extrinsic defect. The identified ground-state structures were further relaxed using a converged 3 × 3 × 3 **k**-point grid. The doped package was then used to postprocess the results and to obtain transition-level diagrams (TLDs), self-consistent Fermi energies, and defect and carrier concentrations. More details about the methodology can be found in the Supporting Information. Our defect calculations followed the recently proposed guidelines for reproducibility^45^ and the data are freely available on Zenodo (10.5281/zenodo.17522862).

## III Electronic Structure, Phonon Dispersion, and Transport Properties

The PBE0 electronic band structure of ZnGa_2_O_4_ is shown in Figure 2(a). The dispersive conduction band minimum (CBM) at Γ results in a low isotropic effective mass of 0.27 *m*_0_, in good agreement with previous works (0.2 to 0.3 *m*_0_).^47,54^ An indirect wide band gap of 5.08 eV is found (5.29 eV for the direct band gap) using standard PBE0 DFT. A more in-depth analysis of the band gap is provided in Section 4. Figure 2(b) shows the DFPT phonon dispersion of ZnGa_2_O_4_. As expected with GGA,^55,56^ the frequencies are slightly underestimated but remain in agreement with experimental measurements.^47–50^ A more detailed analysis of the phonon-mode symmetry and activity is provided in the Supporting Information.

**Figure 2:**
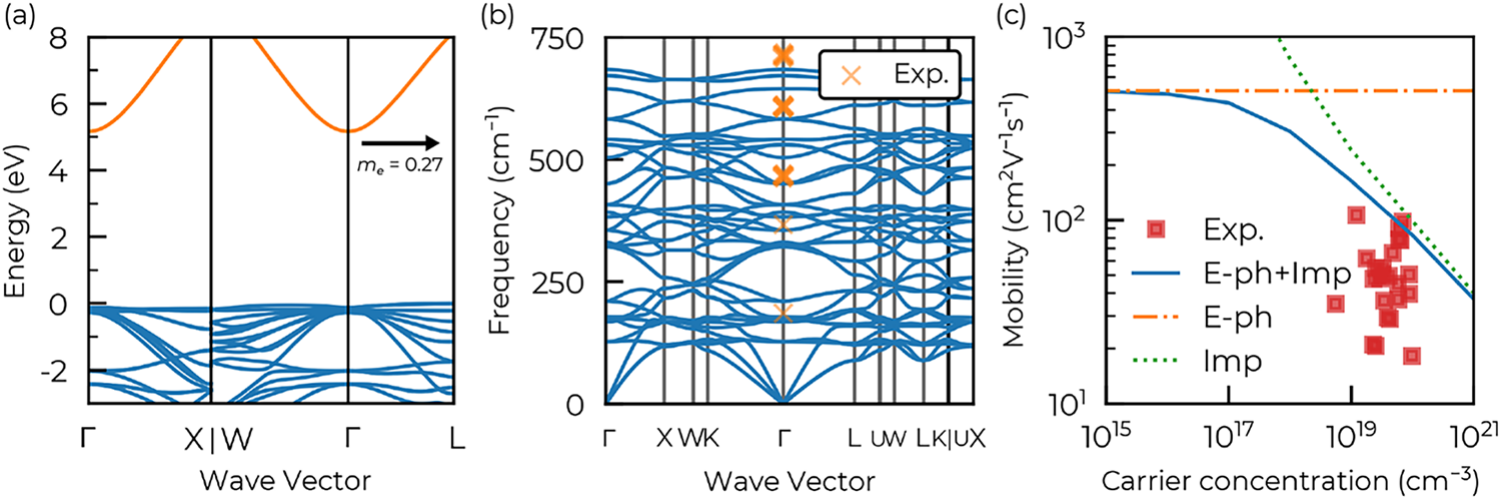
(a) Electronic band structure (plotted using SUMO^46^) and (b) phonon dispersion of ZnGa_2_O_4_. The crosses in the phonon dispersion correspond to experimental measurements reported in different studies.^47–50^ (c) Computed isotropic mobility of ZnGa_2_O_4_ at 300 K split by contributions. E–ph stands for electron–phonon contribution (computed using the iterative Boltzmann transport equation), Imp represents the impurity contribution (computed using a Brooks–Herring model within the Boltzmann transport equation), and E–ph + Imp is our final result combining the two contributions using Matthiessen’s rule. Experimental results are from refs 47,51–53 .

Mobility results as a function of carrier concentrations are listed in Figure 2(c). A phonon-limited mobility (E–ph, in orange in the figure) of 494.6 cm^2^ V^–1^ s^–1^ is found using the exact solution of the BTE. Here, we considered this value as independent of the carrier concentration and it represents an upper limit of the mobility achievable in ZnGa_2_O_4_. On the other hand, the mobility limited by impurity scattering (Imp, in green in the figure) is directly linked to the carrier concentration. The Brooks–Herring model used in this work to model impurity scattering is mainly based on the dielectric constants of ZnGa_2_O_4_. The high-frequency, ϵ_∞_, response computed within the independent particle random phase approximation (IP-RPA) is slightly underestimated with a value of 3.09 with respect to experimental measurements ranging from 3.57 to 3.88 ^6,47,54,57–60^ . Hybrid functionals are in fact often linked to an underestimation of ϵ_∞_ ^35,61^ and a slight overestimation of the scattering is expected.

By combining the two contributions using Matthiessen’s rule, we find our final result (E–ph + Imp, in blue), which closely match the best mobility values achieved experimentally using the vertical gradient freeze (VGF) or Czochralski growth method^6,47,51–53^ . On the one hand, the close agreement with the measured mobilities validates our model, but on the other hand, it suggests only a modest scope for further mobility improvements in experimental samples. In addition, we can also see that impurity scattering is clearly the dominating mechanism in the range of the experimentally observed intrinsic carrier concentrations.

## IV Temperature-Dependent Band Gap

Understanding the temperature dependence of the band gap is essential for predicting accurate electronic and optical properties and, in turn, the derived quantities such as the carrier concentration of defects in this work. However, a complete description is challenging because multiple mechanisms contribute to the temperature evolution of the band gap, including e–ph and e–e interactions, thermal expansion, anharmonic lattice effects, and more.^36–38,62^ In addition, phenomena such as the Burstein–Moss (BM) shift and doping-induced band gap renormalization can further complicate the interpretation of temperature-dependent optical measurements.^3,63^ Here, we restrict our analysis to the renormalization arising from e–ph interactions within the harmonic approximation, as we expected it to capture the major part of the renormalization in ZnGa_2_O_4_, as it is the case in ZnO and Ga_2_O_3_.^36,38,64^

Figure 3 displays the temperature-dependent direct band gap of ZnGa_2_O_4_ (at Γ). At 300 K, we estimate the direct band gap to be around 4.9 eV and the indirect band gap to be around 4.7 eV (assuming that the renormalization affects the band equally). These values compare well with experimental measurements using spectroscopic ellipsometry (between 5.1 and 5.3 eV for the direct band gap and between 4.7 and 5.1 eV for the indirect band gap).^54,60,65^ Using a linear fit, we found a slope of −0.51 meV K^–1^, which is in reasonable agreement with the slope obtained by Hilfiker et al. (−0.72 meV K^–1^). The main part of this discrepancy is probably due to the absence of thermal expansion in our calculations. Thermal expansion has been shown to have a small effect on ZnO (<10%)^36,38^ and is slightly larger for β-Ga_2_O_3_ (around 20%)^64^ and we could expect something intermediate for ZnGa_2_O_4_.

**Figure 3:**
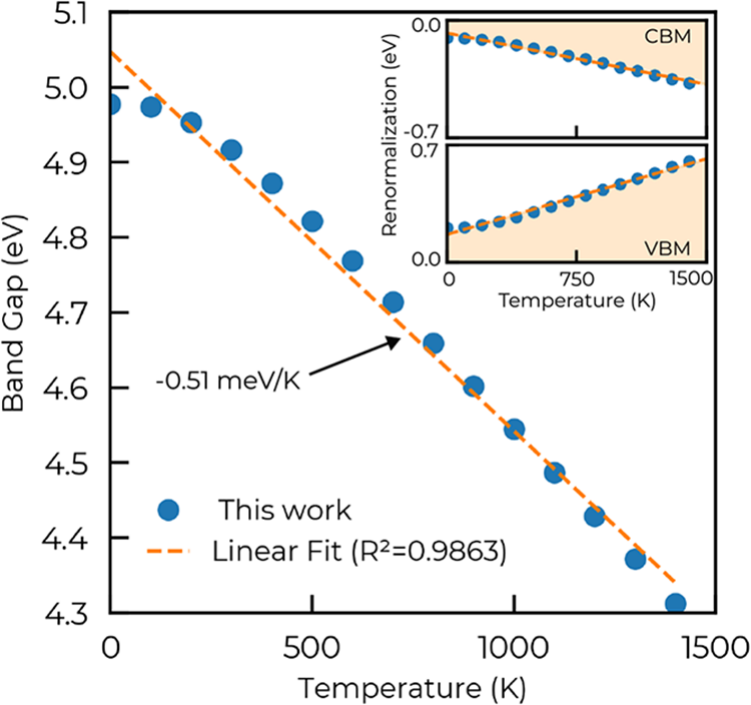
Temperature-dependent direct band gap (at Γ) of ZnGa_2_O_4_. This band gap is obtained by combining the PBE0 standard DFT band gap with the renormalization due to e–ph as computed with DFPT. The insets show the asymmetric renormalization with T of the conduction band minimum and valence band minimum.

Another important insight is the large asymmetry in the renormalization of the band gap, as shown in the inset of Figure 3. The VBM position is affected substantially more than the CBM, by roughly a factor of 2. This difference stems from the (heavy) O-2p VBM in oxides, which couples more strongly to phonons than the more delocalized cation-derived CBM.

## V Defect Chemistry

### V.I Intrinsic Defects

Figure 4(a) presents the formation energies of the intrinsic point defects in ZnGa_2_O_4_ under *n*-type (Ga-rich/O-poor) conditions. Vacancies (*V*_Zn_, *V*_Ga_, and *V*_O_), interstitials (Zn_i_, Ga_i_, and O_i_), and metallic antisites (Zn_Ga_ and Ga_Zn_) were considered. Oxygen–metal antisites were neglected in this study as they were considered unfavorable in ternary oxides,^66^ but also in previous studies on ZnGa_2_O_4_ directly.^12,17,67^ Notably, the interstitial defects can exist in 3 different crystallographically distinct sites: *C*_2*v*_, *T*_*d*_, and *D*_3*d*_. The *T*_*d*_ site was the more energetically favorable site for the three elements and the only one kept for the rest of this work.

**Figure 4:**
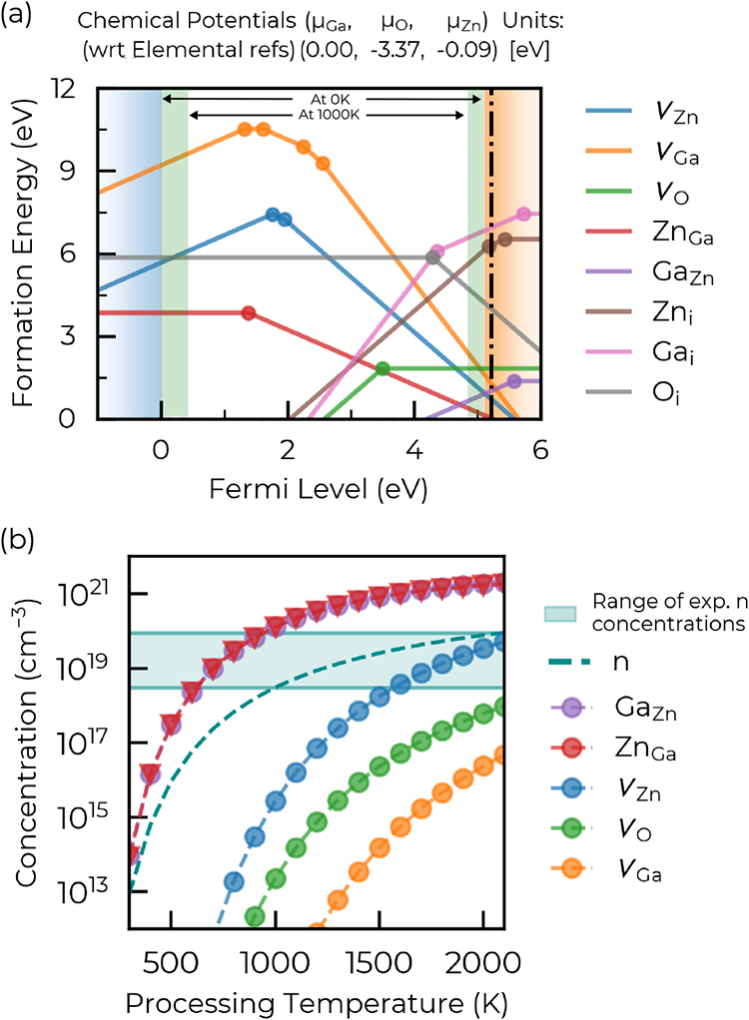
(a) Transition-level diagram of intrinsic point defects in ZnGa_2_O_4_ under Ga-rich/O-poor (*n*-type) conditions. The self-consistent Fermi level (the dotted black line) sits at 5.2 eV at 1000 K. The renormalized band gap at 1000 K is also represented with the light green area. (b) Intrinsic defect and carrier concentrations as a function of the processing temperature. Here, the asymmetric temperature-dependent band gap is taken into account. References 6,10,47,51,68 were used to determine the range of experimental intrinsic electron concentrations. Defects with concentrations lower than 1 × 10^12^ cm^–3^ are omitted for the sake of clarity.

As previously determined,^12,17,67^ the intrinsic defect chemistry in ZnGa_2_O_4_ is rather straightforward: the lowest energy donor defects are Ga_Zn_ and *V*_O_, whereas Zn_Ga_ acts as the most important compensating defect under *n*-type conditions with metallic vacancies (*V*_Zn_, *V*_Ga_) closely following. This is unsurprising, given the fact that it is possible to stabilize the inverse spinel. In fact, the concentrations of both Ga_Zn_ and Zn_Ga_ are directly linked, as shown in Figure 4(b). These two defects pin the Fermi energy and, as such, have effectively equal concentrations (under all conditions). As both cations (Ga^3+^ and Zn^2+^) have similar ionic sizes (∼0.62 and 0.74 Å, respectively), it is not energetically costly to substitute one cation by the other. Finally, the interstitial defects are high in energy due to the closed-packed spinel structure.

Overall, the intrinsic defect landscape in ZnGa_2_O_4_ remains particularly interesting with a self-consistent Fermi level lying above the CBM at 1000 K (the dashed black line in Figure 4(a)). This leads to an intrinsic carrier concentration between 1 × 10^19^ and 1 × 10^20^ cm^–3^ in the typical processing temperature range, as shown in Figure 4(b). This aligns well with the experimentally measured intrinsic carrier concentrations on samples grown using VGF, Czochralski, or metal–organic chemical vapor deposition (MOCVD) methods, ranging from 3 × 10^18^ to 9 × 10^19^ cm^–3^.^6,10,51,68^ Because these synthesis methods are either high-temperature or out-of-equilibrium, they are likely to be consistent with the oxygen-poor conditions identified here. Such high intrinsic carrier concentrations remain rare in oxides and are similar or higher than those in commercialized TCOs such as Ga_2_O_3_.^30,69^ Here, the asymmetric temperature-dependent band gap is key to recover the experimentally observed carrier concentrations. Without any band gap renormalization, the carrier concentration is underestimated by 1 order of magnitude. On the other hand, applying a symmetric effect on both the CBM and the VBM leads to an overestimation of the electron carrier concentration, as the shift of the VBM is twice as large as the shift of the CBM (see the Supporting Information).

Finally, our defect results for *p*-type conditions (O-rich) are provided in the Supporting Information (Figure S5). The Fermi level is deep inside the band gap, which leads to a low hole concentration. Together with recent reports of small-polaron formation,^12,13^ this suggests that intrinsic *p*-type conductivity in ZnGa_2_O_4_ is unlikely.

### V.II Extrinsic Defects

In ZnGa_2_O_4_, the *n*-type doping window offered by the compensating defect Zn_Ga_ is very small (around 0.14 eV), limiting the possibility to increase the *n*-type carrier concentration via donor doping. However, a low-formation-energy dopant could still be of interest to improve the carrier concentration, adding a new source of free carriers to the system. In order to assess if the electron concentration can be improved by external doping, several *n*-type dopants were tried: 4^+^ cations on the Ga site (Si_Ga_, Ge_Ga_, Sn_Ga_, Zr_Ga_, Ti_Ga_, and Hf_Ga_), 3^+^ cations on the Zn site (Al_Zn_, In_Zn_, La_Zn_, Sc_Zn_, and Y_Zn_), and fluorine on the O site (F_O_). The corresponding transition-level diagrams are shown in Figure 5(a,b), where the *n*-type conditions are set to minimize the formation energy of the specific defects (i.e., “dopant”-rich conditions).

**Figure 5:**
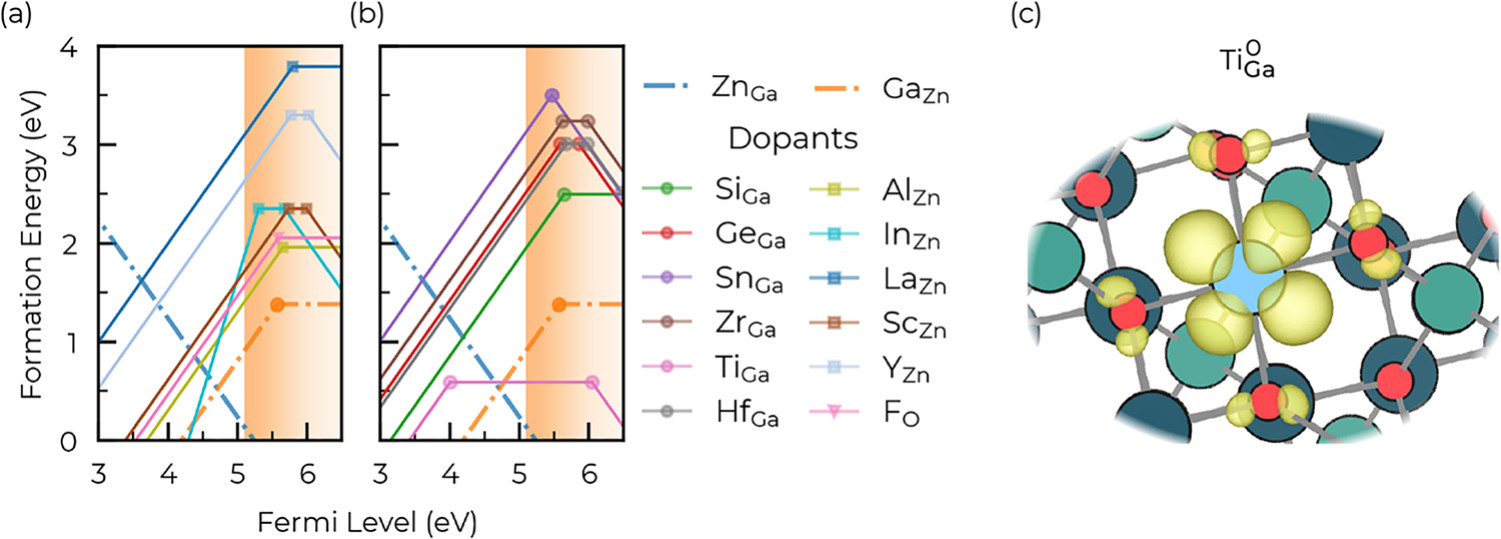
(a) Transition-level diagram of the different extrinsic point defects tested in ZnGa_2_O_4_. Here, we only compute the positive *n*-type defects: 4^+^ cations on the Ga site, (b) 3^+^ cations on the Zn site, and F^–^ on the O site. The *n*-type conditions are set to minimize the formation energy of the specific defects (i.e., “dopant”-rich conditions). Zn_Ga_ and Ga_Zn_ antisites are shown for comparison. (c) Electron density around the Ti_Ga_^0^ low-energy defect.

All of the dopants tested here present high formation energy (or at least a higher formation energy than Ga_Zn_), with the exception of Ti_Ga_. This results in no significant improvement in free electron concentrations as compared to the undoped system. This aligns well with previous experimental results on Ge-, Y-, Si-, and Zr-doped ZnGa_2_O_4_, where no improvements were made due to the introduction of these dopants.^6,30,67,70^ The case of Ti_Ga_ is an outlier; the formation energy is significantly lower than for other dopants but the defect is also deep and associated with a localized charged on Ti, as seen in Figure 5(c). This reflects an electron trapped on Ti, reducing it to Ti­(III).

## VI Conclusion

In summary, we have provided a comprehensive first-principles study of the transport properties, temperature-dependent band gap renormalization, and defect chemistry of ZnGa_2_O_4_. Our transport calculations reproduce state-of-the-art experimental mobilities and demonstrate that phonon-limited mobility can reach nearly 500 cm^2^ V^–1^ s^–1^, although impurity scattering dominates across relevant carrier densities. By explicitly accounting for electron–phonon renormalization of the band gap, we reconcile intrinsic defect calculations with the high electron concentrations (close to 10^20^ cm^–3^) observed experimentally. This asymmetric renormalization, with the valence band shifting nearly twice as strongly as the conduction band, provides new insight into carrier generation in ultrawide-band-gap oxides.

Our defect analysis confirms that Ga_Zn_/Zn_Ga_ antisites govern the intrinsic defect landscape, pinning the Fermi level in the CBM and enabling degenerate *n*-type conductivity, while *p*-type conductivity is unlikely due to deep acceptors and polaron formation. Screening of extrinsic donor dopants reveals little scope for further electron enhancement, as most substitutions either have high formation energies or create deep centers. These findings establish the intrinsic limits of ZnGa_2_O_4_ as a transparent conductor. While it offers exceptional chemical stability, a wide band gap, and high intrinsic mobility, meaningful performance improvements will likely require strategies beyond conventional single-dopant doping, such as nonequilibrium growth, codoping, strain engineering, or heterostructure design. By defining both the potential and the limitations of ZnGa_2_O_4_, this work provides a foundation for exploiting this versatile oxide in next-generation deep-UV optoelectronic and power electronic devices.

## Supplementary Material



## Notes

Details of the defect calculation methodology; phonon analysis; convergence studies for carrier transport and temperature-dependent band gaps; intrinsic *p*-type defect plots; defect formation energy data; and the corresponding Brillouin zone of the structure (PDF)
