# Supplementary material for: Establishing Doping Limits for ZnGa2O4 for Ultrawide-Band-Gap Semiconductor Applications
Source: ACS Appl Mater Interfaces. 2025 Dec 9;17(51):69659–65. doi: 10.1021/acsami.5c19146 (PMC12754759; doi:10.1021/acsami.5c19146)
Supplement: Supplementary file 1 [file am5c19146_si_001.pdf]

Supporting Information - Establishing Doping Limits for $ZnGa_2O_4$ for Ultrawide-Band-gap Semiconductor Applications

Romain Claes ,[1,*] Alexander G. Squires ,[1] and David O. Scanlon [1,†]

[1] *School of Chemistry, University of Birmingham, Edgbaston, Birmingham, B15 2TT, UK*

Supporting Information contains additional details about the defects calculation methodology, a phonon modes analysis, as well as convergence studies for the carrier transport and temperature-dependent bandgap, intrinsic *p*-type defects plots, defect formation energy data and the corresponding Brillouin zone of the structure.

* r.claes@bham.ac.uk

† d.o.scanlon@bham.ac.uk

I. PHONONS ANALYSIS

Following group theory, the cubic structure of $ZnGa_2O_4$ presents 39 optical modes:

$$\Gamma_{\text{opt}} = \underbrace{A_{1g} \oplus E_g \oplus 3T_{2g}}_{\text{Raman}} \oplus \underbrace{4T_{1u}}_{\text{IR}} \oplus \underbrace{T_{1g} \oplus 2A_{2u} \oplus 2E_u \oplus 2T_{2u}}_{\text{-}}$$

Modes	Deg.	Irrep	Activity	This work (cm^{-1})	Experiment (cm^{-1}) [1]	[2]	[3]	[4]
4–6	3	T_{2u}	–	127.4	–	–	–	–
7–9	3	T_{1u}	IR	168.4	–	–	–	–
10–12	3	T_{2g}	Raman	178.4	186	–	–	–
13–14	2	E_u	–	210.2	–	–	–	–
15–17	3	T_{1u}	IR	321.8	–	–	–	–
18–20	3	T_{1g}	–	323.0	–	–	–	–
21–22	2	E_g	Raman	355.5	367	–	–	–
23–25	3	T_{1u}	IR	382.9	–	–	–	–
26	1	A_{2u}	–	392.4	–	–	–	–
27–29	3	T_{2u}	–	407.5	–	–	–	–
30–32	3	T_{2g}	Raman	450.8	468	465	467	469
33–34	2	E_u	–	530.7	–	–	–	–
35–37	3	T_{1u}	IR	541.8	–	–	–	–
38–40	3	T_{2g}	Raman	582.9	610	608	611	609
41	1	A_{2u}	–	672.0	–	–	–	–
42	1	A_{1g}	Raman	684.3	715	710	714	717

TABLE S1. Computed optical Γ-point phonons for spinel (Fd$\bar{3}$m), grouped by degeneracy and by order of frequency.

The Raman modes (observed experimentally in Refs. [1–4] ; corresponding to the crosses in Fig. 2b) correspond to the following vibrational modes in our theoretical analysis.

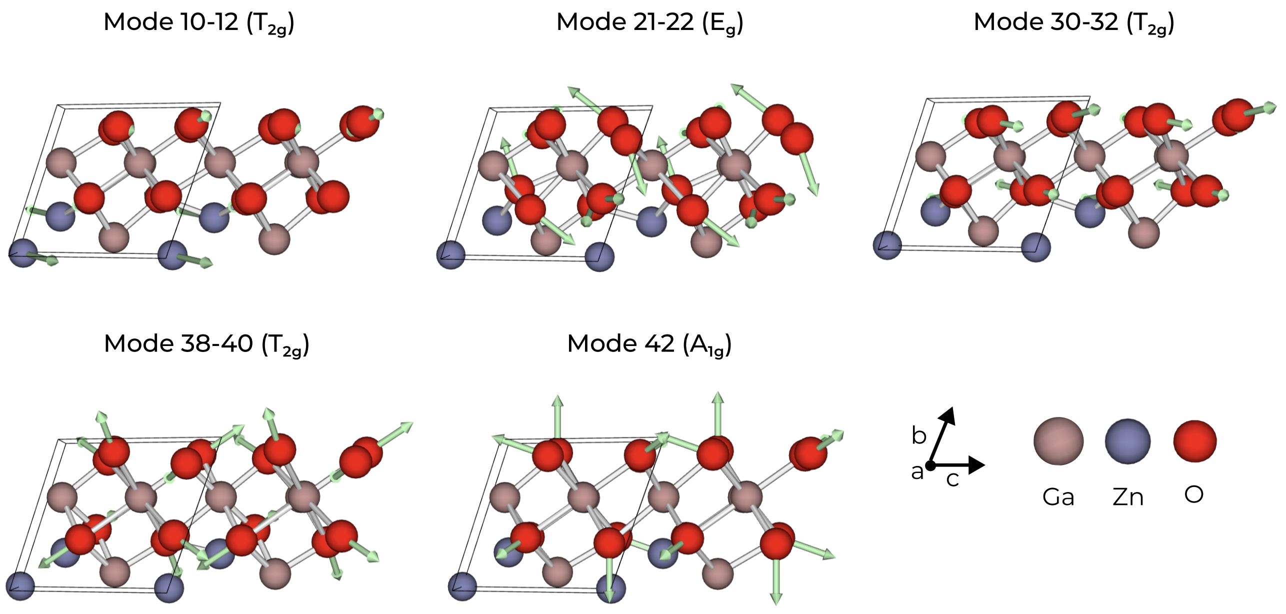

FIG. S1. Illustration of the Raman-active phonons mode.

II. TRANSPORT CALCULATIONS

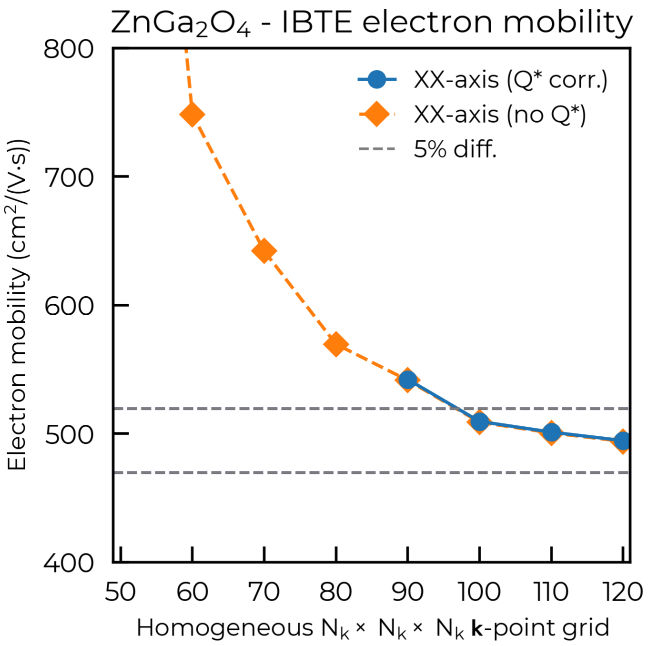

FIG. S2. Convergence of the phonon-limited mobility (iterative Boltzmann transport equation, IBTE) with respect to the k/q-points grids. The results with and without quadrupolar corrections are shown in blue and orange, respectively.

III. TEMPERATURE DEPENDENT BANDGAP CALCULATIONS

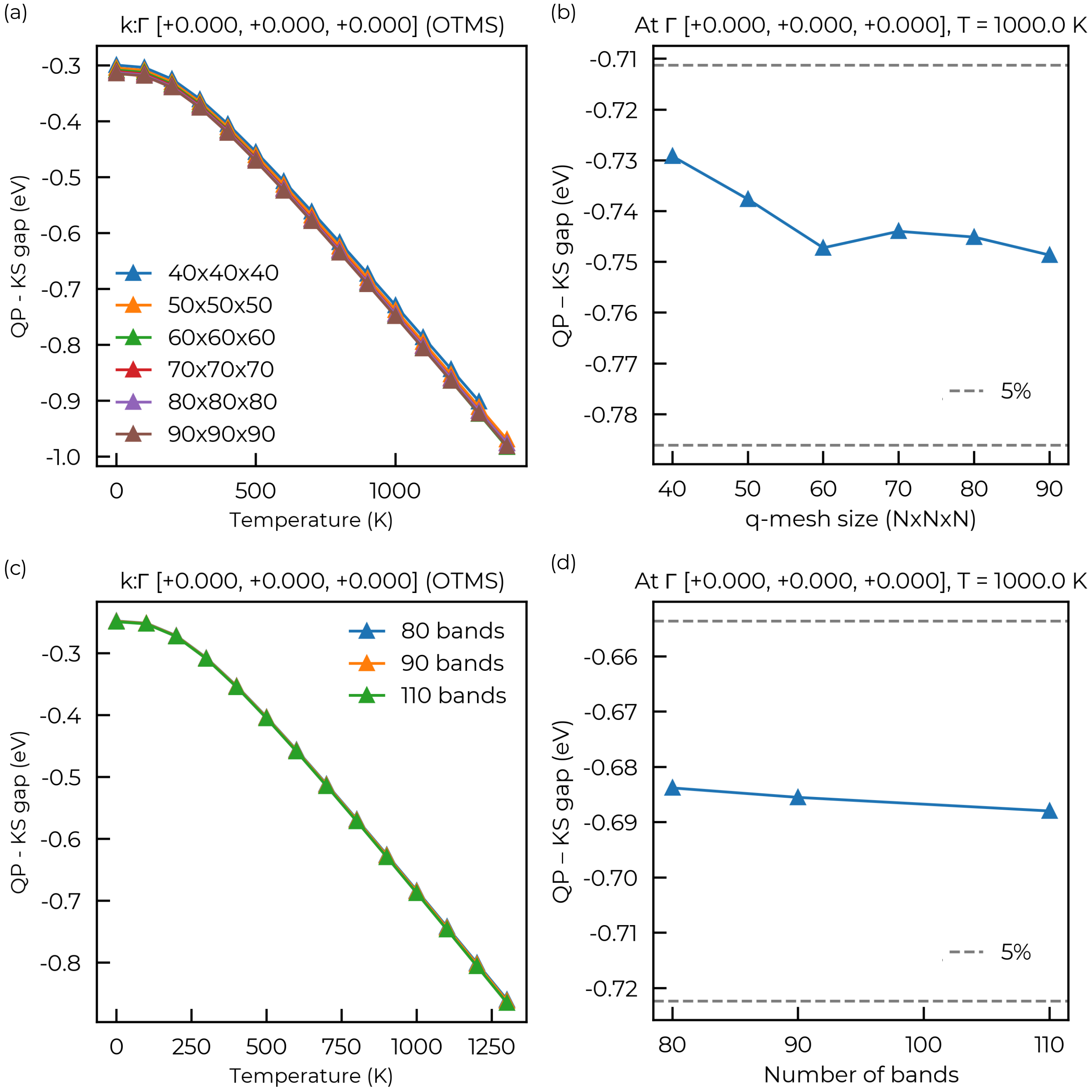

FIG. S3. (a) Convergence of the temperature dependent bandgap (using the on-the-mass-shell approach + Sternheimer method) with the temperature for different k/q-points grids and (b) number of bands. (c) Convergence of the temperature dependent bandgap at 1000K with respect to the k/q-points grids and (d) the number of bands. In this work on $ZnGa_2O_4$, 70 bands are occupied.

IV. DEFECTS CALCULATIONS

A. Formation energy calculations

The formation energy of a point defect X in charge state q is defined by:

$$\Delta E_f^{X^q} = E^{X^q} - E^{\rm bulk} - \sum_i \mu_i + q\, E_{\rm F} + E_{\rm corr}, \tag{1}$$

where E^{X^q} is the total energy of the defective supercell, $E^{\rm bulk}$ is the corresponding pristine supercell energy, i indexes the atomic species with chemical potentials μ_i, q is the defect charge, $E_{\rm F}$ is the Fermi level, and $E_{\rm corr}$ is the finite-size image-charge correction accounting for the use of a finite supercell [5]. In this work, we used the Kumagai-Oba (eFNV) finite-size charge correction scheme [6].

The equilibrium concentrations of charged defects and carriers are determined self-consistently by enforcing charge neutrality, i.e., the total concentration of negatively charged defects and electrons must equal that of positively charged defects and holes:

$$\sum_{X,q} q\,[X^q] + p - n = 0. \tag{2}$$

The concentration of a defect X in charge state q follows Boltzmann statistics and is given by:

$$[X^q] = N_{X^q}\, g_{X^q}\, \exp\left(-\frac{\Delta E_f^{X^q}(E_F, T)}{k_B T}\right), \tag{3}$$

where N_{X^q} is the number of available sites for defect X, g_{X^q} its degeneracy, and $\Delta E_f^{X^q}(E_F, T)$ the formation energy as defined previously.

The equilibrium carrier concentrations are obtained from Fermi–Dirac statistics:

$$n = \int_{E_c}^{\infty} D_c(E)\, f(E, E_F, T)\, \mathrm{d}E, \tag{4}$$

$$p = \int_{-\infty}^{E_v} D_v(E)\, [1 - f(E, E_F, T)]\, \mathrm{d}E, \tag{5}$$

where $D_c(E)$ and $D_v(E)$ are the density of states of the conduction and valence bands, respectively, and $f(E, E_F, T)$ is the Fermi–Dirac distribution function:

$$f(E, E_F, T) = \frac{1}{1 + \exp\left(\frac{E - E_F}{k_B T}\right)}. \tag{6}$$

The Fermi energy E_F is determined iteratively such that the charge-neutrality condition (Eq. 2) is satisfied at the chosen temperature. Defect calculation setup and analysis were performed using the Doped [7] and ShakeNBreak [8, 9] packages.

B. Chemical potentials limits

The PBE0-calculated accessible range of chemical potentials for $ZnGa_2O_4$ is illustrated in Figure S3. The shaded region represents the stable range of chemical potentials ($\Delta\mu_{\rm Ga}$, $\Delta\mu_{\rm Zn}$, and $\Delta\mu_{\rm O}$) imposed by the formation of $ZnGa_2O_4$ and constrained by competing phases (Ga_2O_3, ZnO, and O_2) [10]. The thermodynamic stability of $ZnGa_2O_4$ requires that

$$\Delta\mu_{\rm Zn} + 2\,\Delta\mu_{\rm Ga} + 4\,\Delta\mu_{\rm O} = \Delta H_f(\mathrm{ZnGa_2O_4}), \tag{7}$$

$$\Delta\mu_{\rm Zn} \leq 0, \tag{8}$$

$$\Delta\mu_{\rm Ga} \leq 0, \tag{9}$$

$$\Delta\mu_{\rm O} \leq 0, \tag{10}$$

and, to prevent precipitation of competing phases,

$$2\,\Delta\mu_{\mathrm{Ga}} + 3\,\Delta\mu_{\mathrm{O}} \le \Delta H_f(\mathrm{Ga_2O_3}), \tag{11}$$

$$\Delta\mu_{\mathrm{Zn}} + \Delta\mu_{\mathrm{O}} \le \Delta H_f(\mathrm{ZnO}), \tag{12}$$

$$\Delta\mu_{\mathrm{O}} \le \tfrac{1}{2}\Delta H_f(\mathrm{O_2}) = 0. \tag{13}$$

Within these boundaries, the red dot in Figure S3 marks the chosen n-type growth conditions (metal-rich, oxygen-poor) used in this study.

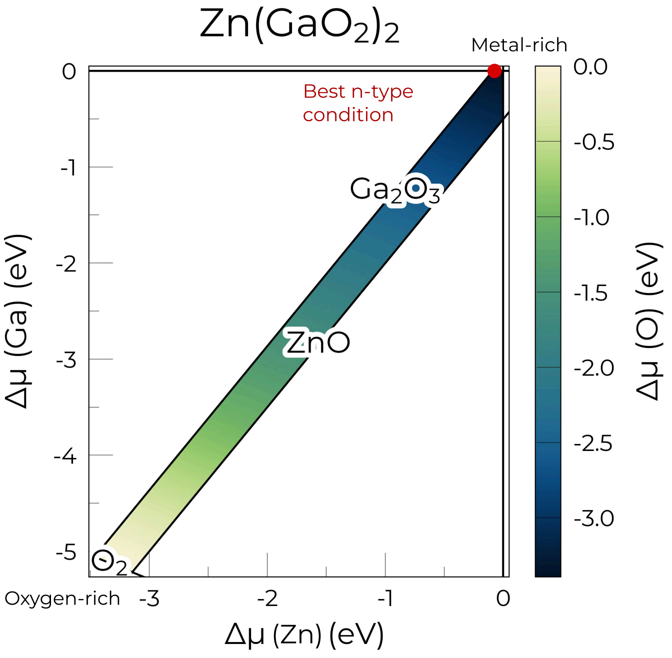

FIG. S4. Illustration of the accessible chemical potential range.

C. Additional defect plots

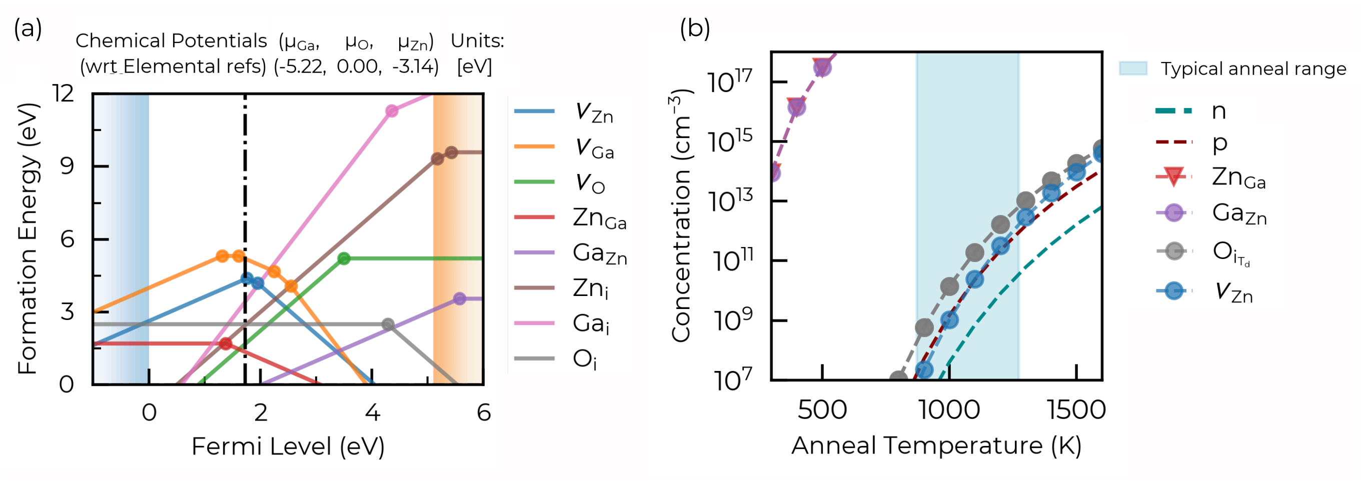

FIG. S5. (a) Transition level diagram of intrinsic point defects in $ZnGa_2O_4$ under O-rich/Ga-poor (p-type) conditions. The self-consistent Fermi level (dotted black line) sits at 1.7 eV above the valence band minimum (at 1000 K). (b) Intrinsic defect and carrier concentrations as a function of annealing temperature. Here, the asymmetric temperature-dependent bandgap is taken into account.

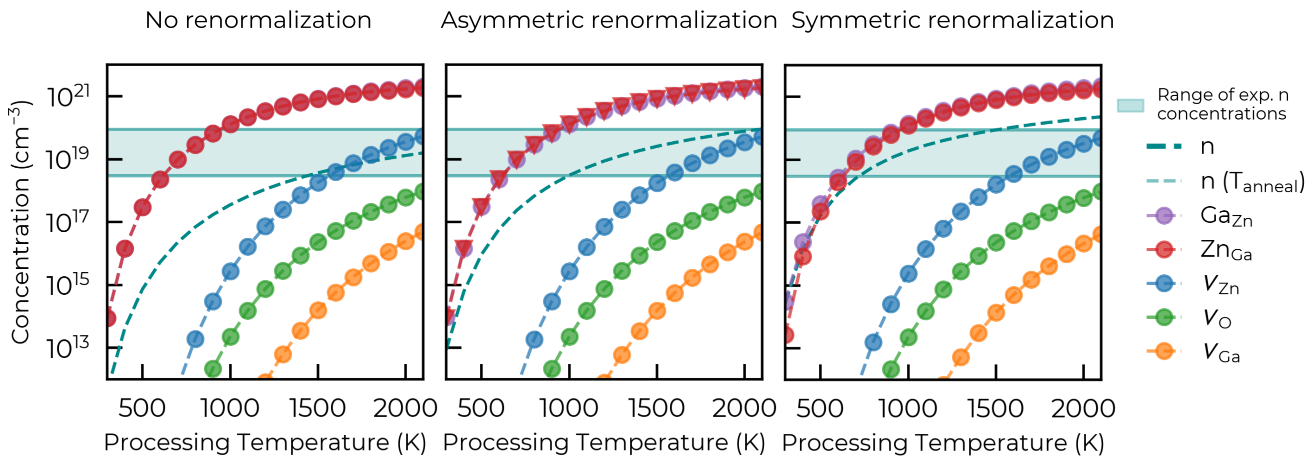

FIG. S6. Defect and carrier concentrations with different type of temperature-dependent bandgap. Using the asymmetric renormalization of the bandgap is key to recover experimental hole densities whereas a symmetric renormalization tends to overestimate the carrier concentration.

D. Intrinsic Defect Chemistry datas

Formula	Space Group	E_{Hull} (eV/atom)	k-mesh	ΔE_f (eV/fu)
$Zn(GaO_2)_2$	$Fd\bar{3}m$	0.000	5×5×5	-13.583
Zn	$P6_3/mmc$	0.000	13×13×7	0.000
Ga	Cmce	0.000	6×6×5	0.000
O_2	Pmmm	0.000	1×1×1	0.000
ZnO	$P6_3mc$	0.000	6×6×4	-3.144
Ga_2O_3	C2/m	0.000	4×4×4	-10.120

TABLE S2. Formation energies per formula unit (ΔE_f) of $ZnGa_2O_4$ and all competing phases, with k-meshes used in calculations. Only the lowest energy polymorphs are included.

E. Extrinsic Defect Chemistry datas

F. Al-doped $ZnGa_2O_4$

Formula	Space Group	E_{Hull} (eV/atom)	k-mesh	ΔE_f (eV/fu)
$Zn(GaO_2)_2$	$Fd\bar{3}m$	0.000	5×5×5	-13.583
Zn	$P6_3/mmc$	0.000	13×13×7	0.000
Al	$Fm\bar{3}m$	0.000	18×18×18	0.000
Ga	Cmce	0.000	6×6×5	0.000
O_2	Pmmm	0.000	1×1×1	0.000
ZnO	$P6_3mc$	0.000	6×6×4	-3.144
Ga_2O_3	C2/m	0.000	4×4×4	-10.120
Al_2ZnO_4	$Fd\bar{3}m$	0.000	3×3×3	-19.733

TABLE S3. Formation energies per formula unit (ΔE_f) of Al-doped $ZnGa_2O_4$ and all competing phases, with k-meshes used in calculations. Only the lowest energy polymorphs are included.

Limit	Zn	Ga	O	Al	Al-Limiting Phase
$Zn(GaO_2)_2$–Ga_2O_3–Ga	-0.089	0.000	-3.373	-3.075	Al_2ZnO_4
$Zn(GaO_2)_2$–Zn–Ga	0.000	0.000	-3.396	-3.075	Al_2ZnO_4
$Zn(GaO_2)_2$–Zn–ZnO	0.000	-0.503	-3.144	-3.578	Al_2ZnO_4
$Zn(GaO_2)_2$–O_2–ZnO	-3.144	-5.219	0.000	-8.294	Al_2ZnO_4
$Zn(GaO_2)_2$–Ga_2O_3–O_2	-3.463	-5.060	0.000	-8.135	Al_2ZnO_4

TABLE S4. Chemical potential limits for Al-doped $ZnGa_2O_4$.

G. F-doped $ZnGa_2O_4$

Formula	Space Group	E_{Hull} (eV/atom)	k-mesh	ΔE_f (eV/fu)
$Zn(GaO_2)_2$	$Fd\bar{3}m$	0.000	5×5×5	-13.583
Zn	$P6_3/mmc$	0.000	13×13×7	0.000
Ga	Cmce	0.000	6×6×5	0.000
O_2	Pmmm	0.000	1×1×1	0.000
F_2	Pmmm	0.000	1×1×1	0.000
ZnO	$P6_3mc$	0.000	6×6×4	-3.144
ZnF_2	$P4_2/mnm$	0.000	3×3×4	-7.542
Ga_2O_3	C2/m	0.000	4×4×4	-10.120
GaF_3	$R\bar{3}c$	0.000	3×3×3	-11.236

TABLE S5. Formation energies per formula unit (ΔE_f) of F-doped $ZnGa_2O_4$ and all competing phases, with k-meshes used in calculations. Only the lowest energy polymorphs are included.

Limit	Zn	Ga	O	F	F-Limiting Phase
$Zn(GaO_2)_2$–Ga_2O_3–Ga	-0.089	0.000	-3.373	-3.745	GaF_3
$Zn(GaO_2)_2$–Zn–Ga	0.000	0.000	-3.396	-3.771	ZnF_2
$Zn(GaO_2)_2$–Zn–ZnO	0.000	-0.503	-3.144	-3.771	ZnF_2
$Zn(GaO_2)_2$–O_2–ZnO	-3.144	-5.219	0.000	-2.199	ZnF_2
$Zn(GaO_2)_2$–Ga_2O_3–O_2	-3.463	-5.060	0.000	-2.059	GaF_3

TABLE S6. Chemical potential limits for F-doped $ZnGa_2O_4$.

H. Ge-doped $ZnGa_2O_4$

Formula	Space Group	E_{Hull} (eV/atom)	k-mesh	ΔE_f (eV/fu)
$Zn(GaO_2)_2$	$Fd\overline{3}m$	0.000	5×5×5	-13.583
Zn	$P6_3/mmc$	0.000	13×13×7	0.000
Ga	Cmce	0.000	6×6×5	0.000
Ge	$Fd\overline{3}m$	0.000	12×12×12	0.000
O_2	Pmmm	0.000	1×1×1	0.000
ZnO	$P6_3mc$	0.000	6×6×4	-3.144
Ga_2O_3	C2/m	0.000	4×4×4	-10.120
Zn_2GeO_4	$R\overline{3}$	0.000	2×2×2	-12.041
Ga_4GeO_8	C2/m	0.000	5×5×3	-25.580

TABLE S7. Formation energies per formula unit (ΔE_f) of Ge-doped $ZnGa_2O_4$ and all competing phases, with k-meshes used in calculations. Only the lowest energy polymorphs are included.

Limit	Zn	Ga	O	Ge	Ge-Limiting Phase
$Zn(GaO_2)_2$–Ga_2O_3–Ga	-0.089	0.000	-3.373	0.000	Ge
$Zn(GaO_2)_2$–Zn–Ga	0.000	0.000	-3.396	0.000	Ge
$Zn(GaO_2)_2$–Zn–ZnO	0.000	-0.503	-3.144	0.000	Ge
$Zn(GaO_2)_2$–O_2–ZnO	-3.144	-5.219	0.000	-5.753	Zn_2GeO_4
$Zn(GaO_2)_2$–Ga_2O_3–O_2	-3.463	-5.060	0.000	-5.340	Ga_4GeO_8

TABLE S8. Chemical potential limits for Ge-doped $ZnGa_2O_4$.

I. In-doped $ZnGa_2O_4$

Formula	Space Group	E_{Hull} (eV/atom)	k-mesh	ΔE_f (eV/fu)
$Zn(GaO_2)_2$	$Fd\overline{3}m$	0.000	5×5×5	-13.583
Zn	$P6_3/mmc$	0.000	13×13×7	0.000
Ga	Cmce	0.000	6×6×5	0.000
In	$R\overline{3}m$	0.000	11×11×11	0.000
O_2	Pmmm	0.000	1×1×1	0.000
ZnO	$P6_3mc$	0.000	6×6×4	-3.144
Ga_2O_3	C2/m	0.000	4×4×4	-10.120
In_2O_3	$Ia\overline{3}$	0.000	2×2×2	-8.615

TABLE S9. Formation energies per formula unit (ΔE_f) of In-doped $ZnGa_2O_4$ and all competing phases, with k-meshes used in calculations. Only the lowest energy polymorphs are included.

Limit	Zn	Ga	O	In	In-Limiting Phase
$Zn(GaO_2)_2$–Ga_2O_3–Ga	-0.089	0.000	-3.373	0.000	In
$Zn(GaO_2)_2$–Zn–Ga	0.000	0.000	-3.396	0.000	In
$Zn(GaO_2)_2$–Zn–ZnO	0.000	-0.503	-3.144	0.000	In
$Zn(GaO_2)_2$–O_2–ZnO	-3.144	-5.219	0.000	-4.308	In_2O_3
$Zn(GaO_2)_2$–Ga_2O_3–O_2	-3.463	-5.060	0.000	-4.308	In_2O_3

TABLE S10. Chemical potential limits for In-doped $ZnGa_2O_4$.

J. Si-doped $ZnGa_2O_4$

Formula	Space Group	E_{Hull} (eV/atom)	k-mesh	ΔE_f (eV/fu)
$Zn(GaO_2)_2$	$Fd\overline{3}m$	0.000	5×5×5	-13.583
Zn	$P6_3/mmc$	0.000	13×13×7	0.000
Ga	Cmce	0.000	6×6×5	0.000
Si	$Fd\overline{3}m$	0.000	8×8×8	0.000
O_2	Pmmm	0.000	1×1×1	0.000
ZnO	$P6_3mc$	0.000	6×6×4	-3.144
Ga_2O_3	C2/m	0.000	4×4×4	-10.120
SiO_2	$I\overline{4}2d$	0.000	3×3×3	-8.958
Zn_2SiO_4	$R\overline{3}$	0.000	2×2×2	-15.534

TABLE S11. Formation energies per formula unit (ΔE_f) of Si-doped $ZnGa_2O_4$ and all competing phases, with k-meshes used in calculations. Only the lowest energy polymorphs are included.

Limit	Zn	Ga	O	Si	Si-Limiting Phase
$Zn(GaO_2)_2$–Ga_2O_3–Ga	-0.089	0.000	-3.373	-2.211	SiO_2
$Zn(GaO_2)_2$–Zn–Ga	0.000	0.000	-3.396	-2.167	SiO_2
$Zn(GaO_2)_2$–Zn–ZnO	0.000	-0.503	-3.144	-2.956	Zn_2SiO_4
$Zn(GaO_2)_2$–O_2–ZnO	-3.144	-5.219	0.000	-9.245	Zn_2SiO_4
$Zn(GaO_2)_2$–Ga_2O_3–O_2	-3.463	-5.060	0.000	-8.958	SiO_2

TABLE S12. Chemical potential limits for Si-doped $ZnGa_2O_4$.

K. Sn-doped $ZnGa_2O_4$

Formula	Space Group	E_{Hull} (eV/atom)	k-mesh	ΔE_f (eV/fu)
$Zn(GaO_2)_2$	$Fd\overline{3}m$	0.000	5×5×5	-13.583
Zn	$P6_3/mmc$	0.000	13×13×7	0.000
Ga	Cmce	0.000	6×6×5	0.000
Sn	$Fd\overline{3}m$	0.000	11×11×11	0.000
O_2	Pmmm	0.000	1×1×1	0.000
ZnO	$P6_3mc$	0.000	6×6×4	-3.144
Ga_2O_3	C2/m	0.000	4×4×4	-10.120
SnO_2	$P4_2/mnm$	0.000	4×4×6	-5.275

TABLE S13. Formation energies per formula unit (ΔE_f) of Sn-doped $ZnGa_2O_4$ and all competing phases, with k-meshes used in calculations. Only the lowest energy polymorphs are included.

Limit	Zn	Ga	O	Sn	Sn-Limiting Phase
$Zn(GaO_2)_2$–Ga_2O_3–Ga	-0.089	0.000	-3.373	0.000	Sn
$Zn(GaO_2)_2$–Zn–Ga	0.000	0.000	-3.396	0.000	Sn
$Zn(GaO_2)_2$–Zn–ZnO	0.000	-0.503	-3.144	0.000	Sn
$Zn(GaO_2)_2$–O_2–ZnO	-3.144	-5.219	0.000	-5.275	SnO_2
$Zn(GaO_2)_2$–Ga_2O_3–O_2	-3.463	-5.060	0.000	-5.275	SnO_2

TABLE S14. Chemical potential limits for Sn-doped $ZnGa_2O_4$.

L. Ti-doped $ZnGa_2O_4$

Formula	Space Group	E_{Hull} (eV/atom)	k-mesh	ΔE_f (eV/fu)
$Zn(GaO_2)_2$	$Fd\bar{3}m$	0.000	5×5×5	-13.583
Ti	P6/mmm	0.000	4×4×7	0.000
Zn	$P6_3/mmc$	0.000	13×13×7	0.000
Ga	Cmce	0.000	6×6×5	0.000
O_2	Pmmm	0.000	1×1×1	0.000
TiO_2	C2/m	0.000	4×4×3	-9.695
ZnO	$P6_3mc$	0.000	6×6×4	-3.144
Ga_2O_3	C2/m	0.000	4×4×4	-10.120
$Ti_3Zn_2O_8$	$P4_332$	0.000	2×2×2	-35.597

TABLE S15. Formation energies per formula unit (ΔE_f) of Ti-doped $ZnGa_2O_4$ and all competing phases, with k-meshes used in calculations. Only the lowest energy polymorphs are included.

Limit	Zn	Ga	O	Ti	Ti-Limiting Phase
$Zn(GaO_2)_2$–Ga_2O_3–Ga	-0.089	0.000	-3.373	-2.948	TiO_2
$Zn(GaO_2)_2$–Zn–Ga	0.000	0.000	-3.396	-2.904	TiO_2
$Zn(GaO_2)_2$–Zn–ZnO	0.000	-0.503	-3.144	-3.481	$Ti_3Zn_2O_8$
$Zn(GaO_2)_2$–O_2–ZnO	-3.144	-5.219	0.000	-9.769	$Ti_3Zn_2O_8$
$Zn(GaO_2)_2$–Ga_2O_3–O_2	-3.463	-5.060	0.000	-9.695	TiO_2

TABLE S16. Chemical potential limits for Ti-doped $ZnGa_2O_4$.

M. Hf-doped $ZnGa_2O_4$

Formula	Space Group	E_{Hull} (eV/atom)	k-mesh	ΔE_f (eV/fu)
$Zn(GaO_2)_2$	$Fd\bar{3}m$	0.000	5×5×5	-13.583
Hf	$P6_3/mmc$	0.000	11×7×7	0.000
Zn	$P6_3/mmc$	0.000	13×13×7	0.000
Ga	Cmce	0.000	6×6×5	0.000
O_2	Pmmm	0.000	1×1×1	0.000
HfO_2	$P2_1/c$	0.000	3×3×3	-11.295
ZnO	$P6_3mc$	0.000	6×6×4	-3.144
Ga_2O_3	C2/m	0.000	4×4×4	-10.120

TABLE S17. Formation energies per formula unit (ΔE_f) of Hf-doped $ZnGa_2O_4$ and all competing phases, with k-meshes used in calculations. Only the lowest energy polymorphs are included.

Limit	Zn	Ga	O	Hf	Hf-Limiting Phase
$Zn(GaO_2)_2$–Ga_2O_3–Ga	-0.089	0.000	-3.373	-4.548	HfO_2
$Zn(GaO_2)_2$–Zn–Ga	0.000	0.000	-3.396	-4.503	HfO_2
$Zn(GaO_2)_2$–Zn–ZnO	0.000	-0.503	-3.144	-5.006	HfO_2
$Zn(GaO_2)_2$–O_2–ZnO	-3.144	-5.219	0.000	-11.295	HfO_2
$Zn(GaO_2)_2$–Ga_2O_3–O_2	-3.463	-5.060	0.000	-11.295	HfO_2

TABLE S18. Chemical potential limits for Hf-doped $ZnGa_2O_4$.

N. La-doped $ZnGa_2O_4$

Formula	Space Group	E_{Hull} (eV/atom)	k-mesh	ΔE_f (eV/fu)
$Zn(GaO_2)_2$	$Fd\overline{3}m$	0.000	5×5×5	-13.583
La	$P6_3/mmc$	0.000	9×9×2	0.000
Zn	$P6_3/mmc$	0.000	13×13×7	0.000
Ga	Cmce	0.000	6×6×5	0.000
O_2	Pmmm	0.000	1×1×1	0.000
ZnO	$P6_3mc$	0.000	6×6×4	-3.144
Ga_2O_3	C2/m	0.000	4×4×4	-10.120
$La_3Ga_5O_{12}$	$Ia\overline{3}d$	0.000	2×2×2	-53.777
$La_4Ga_2O_9$	$P2_1/c$	0.000	2×2×1	-47.677

TABLE S19. Formation energies per formula unit (ΔE_f) of La-doped $ZnGa_2O_4$ and all competing phases, with k-meshes used in calculations. Only the lowest energy polymorphs are included.

Limit	Zn	Ga	O	La	La-Limiting Phase
$Zn(GaO_2)_2$–Ga_2O_3–Ga	-0.089	0.000	-3.373	-4.432	$La_3Ga_5O_{12}$
$Zn(GaO_2)_2$–Zn–Ga	0.000	0.000	-3.396	-4.343	$La_3Ga_5O_{12}$
$Zn(GaO_2)_2$–Zn–ZnO	0.000	-0.503	-3.144	-4.593	$La_4Ga_2O_9$
$Zn(GaO_2)_2$–O_2–ZnO	-3.144	-5.219	0.000	-9.310	$La_4Ga_2O_9$
$Zn(GaO_2)_2$–Ga_2O_3–O_2	-3.463	-5.060	0.000	-9.492	$La_3Ga_5O_{12}$

TABLE S20. Chemical potential limits for La-doped $ZnGa_2O_4$.

O. Sc-doped $ZnGa_2O_4$

Formula	Space Group	E_{Hull} (eV/atom)	k-mesh	ΔE_f (eV/fu)
$Zn(GaO_2)_2$	$Fd\overline{3}m$	0.000	5×5×5	-13.583
Sc	$P6_3/mmc$	0.000	9×9×5	0.000
Zn	$P6_3/mmc$	0.000	13×13×7	0.000
Ga	Cmce	0.000	6×6×5	0.000
O_2	Pmmm	0.000	1×1×1	0.000
Sc_2O_3	$Ia\overline{3}$	0.000	2×2×2	-18.905
ZnO	$P6_3mc$	0.000	6×6×4	-3.144
Ga_2O_3	C2/m	0.000	4×4×4	-10.120

TABLE S21. Formation energies per formula unit (ΔE_f) of Sc-doped $ZnGa_2O_4$ and all competing phases, with k-meshes used in calculations. Only the lowest energy polymorphs are included.

Limit	Zn	Ga	O	Sc	Sc-Limiting Phase
$Zn(GaO_2)_2$–Ga_2O_3–Ga	-0.089	0.000	-3.373	-4.392	Sc_2O_3
$Zn(GaO_2)_2$–Zn–Ga	0.000	0.000	-3.396	-4.359	Sc_2O_3
$Zn(GaO_2)_2$–Zn–ZnO	0.000	-0.503	-3.144	-4.736	Sc_2O_3
$Zn(GaO_2)_2$–O_2–ZnO	-3.144	-5.219	0.000	-9.453	Sc_2O_3
$Zn(GaO_2)_2$–Ga_2O_3–O_2	-3.463	-5.060	0.000	-9.453	Sc_2O_3

TABLE S22. Chemical potential limits for Sc-doped $ZnGa_2O_4$.

P. Y-doped $ZnGa_2O_4$

Formula	Space Group	E_{Hull} (eV/atom)	k-mesh	ΔE_f (eV/fu)
$Zn(GaO_2)_2$	$Fd\overline{3}m$	0.000	5×5×5	-13.583
Y	$R\overline{3}m$	0.000	9×9×9	0.000
Zn	$P6_3/mmc$	0.000	13×13×7	0.000
Ga	Cmce	0.000	6×6×5	0.000
O_2	Pmmm	0.000	1×1×1	0.000
ZnO	$P6_3mc$	0.000	6×6×4	-3.144
Ga_2O_3	C2/m	0.000	4×4×4	-10.120
$Y_3Ga_5O_{12}$	$Ia\overline{3}d$	0.000	2×2×2	-54.817

TABLE S23. Formation energies per formula unit (ΔE_f) of Y-doped $ZnGa_2O_4$ and all competing phases, with k-meshes used in calculations. Only the lowest energy polymorphs are included.

Limit	Zn	Ga	O	Y	Y-Limiting Phase
$Zn(GaO_2)_2$–Ga_2O_3–Ga	-0.089	0.000	-3.373	-4.778	$Y_3Ga_5O_{12}$
$Zn(GaO_2)_2$–Zn–Ga	0.000	0.000	-3.396	-4.689	$Y_3Ga_5O_{12}$
$Zn(GaO_2)_2$–Zn–ZnO	0.000	-0.503	-3.144	-4.857	$Y_3Ga_5O_{12}$
$Zn(GaO_2)_2$–O_2–ZnO	-3.144	-5.219	0.000	-9.573	$Y_3Ga_5O_{12}$
$Zn(GaO_2)_2$–Ga_2O_3–O_2	-3.463	-5.060	0.000	-9.839	$Y_3Ga_5O_{12}$

TABLE S24. Chemical potential limits for Y-doped $ZnGa_2O_4$.

Q. Zr-doped $ZnGa_2O_4$

Formula	Space Group	E_{Hull} (eV/atom)	k-mesh	ΔE_f (eV/fu)
$Zn(GaO_2)_2$	Fd$\bar{3}$m	0.000	5×5×5	-13.583
Zr	$P6_3/mmc$	0.000	8×8×5	0.000
Zn	$P6_3/mmc$	0.000	13×13×7	0.000
Ga	Cmce	0.000	6×6×5	0.000
O_2	Pmmm	0.000	1×1×1	0.000
ZrO_2	$P2_1/c$	0.000	3×3×3	-10.680
ZnO	$P6_3mc$	0.000	6×6×4	-3.144
Ga_2O_3	C2/m	0.000	4×4×4	-10.120

TABLE S25. Formation energies per formula unit (ΔE_f) of Zr-doped $ZnGa_2O_4$ and all competing phases, with k-meshes used in calculations. Only the lowest energy polymorphs are included.

V. ADDITIONAL FIGURES

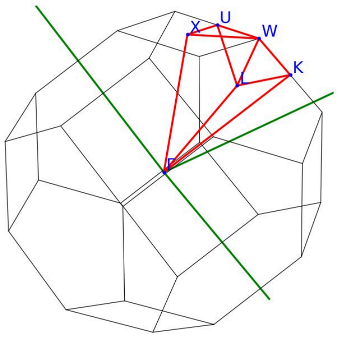

FIG. S7. Brillouin zone with the corresponding k/q-path used for the electronic and phonon band structures.

[1] A. Wüthrich, R. Goldhahn, Z. Galazka, and M. Feneberg, Phys. Rev. Mater. **9**, 064602 (2025).
[2] S. López-Moreno, P. Rodríguez-Hernández, A. Muñoz, A. Romero, F. Manjón, D. Errandonea, E. Rusu, and V. Ursaki, Ann. Phys. **523**, 157–167 (2011).
[3] G. G. P. Van Gorkom, J. H. Haanstra, and H. v. d. Boom, J. Raman Spectrosc. **1**, 513–519 (1973).
[4] Z. Shi, S. Li, Z. Zheng, X. Feng, Z. Fang, J. Yang, and B. Tang, J. Electron. Mater. **53**, 2240–2249 (2024).
[5] S. Zhang and J. Northrup, Phys. Rev. Lett. **67**, 2339–2342 (1991).
[6] Y. Kumagai and F. Oba, Phys. Rev. B **89**, 195205 (2014).
[7] S. R. Kavanagh, A. G. Squires, A. Nicolson, I. Mosquera-Lois, A. M. Ganose, B. Zhu, K. Brlec, A. Walsh, and D. O. Scanlon, J. Open Source Softw. **9**, 6433 (2024).
[8] I. Mosquera-Lois, S. R. Kavanagh, A. Walsh, and D. O. Scanlon, J. Open Source Softw. **7**, 4817 (2022).
[9] I. Mosquera-Lois, S. R. Kavanagh, A. Walsh, and D. O. Scanlon, npj Comput. Mater. **9**, 25 (2023).
[10] J. Buckeridge, D. Scanlon, A. Walsh, and C. Catlow, Comput. Phys. Commun. **185**, 330–338 (2014).
